# “I feel like a fish out of water”: interpreting the occupational stress and well-being experiences of professional classical musicians

**DOI:** 10.3389/fpsyg.2024.1374773

**Published:** 2024-08-14

**Authors:** Simone Willis, Mikel Mellick, Rich Neil, David Wasley

**Affiliations:** ^1^Cardiff School of Sport and Health Sciences, Cardiff Metropolitan University, Cardiff, United Kingdom; ^2^Specialist Unit for Review Evidence, Cardiff University, Neuadd Meirionnydd, Heath Park, Cardiff, United Kingdom; ^3^Centre for Health, Activity and Wellbeing Research (CAWR), School of Sport and Health Sciences, Cardiff Metropolitan University, Cardiff, United Kingdom; ^4^Research and Innovation Services, Cardiff Metropolitan University, Cardiff, United Kingdom

**Keywords:** performance, musicians, stress, demands, appraisal, coping, well-being, interpretative phenomenological analysis

## Abstract

**Introduction:**

Professional classical musicians operate within a highly demanding environment, which includes organizational, social, and emotional demands. When not effectively coped with, these demands may cause stress and negatively impact well-being. This qualitative study explored the perceived stress and well-being experiences of professional classical musicians through a transactional theory of stress. The study employed a double hermeneutic interpretation of the lived experiences of the perceived demands faced, stress appraisals made, resources used, and the influence on well-being.

**Methods:**

Six professional classical musicians were purposefully selected for participation. Semi-structured interviews were conducted and participants reflected on two events: one they perceived as a positive experience and one that was negative. Transcripts were analyzed using Interpretative Phenomenological Analysis and Group Experiential Themes emerged.

**Results:**

Three Group Experiential Themes were identified: (a) Performance Demands; (b) Organizational Demands; and, (c) Relationship Demands. Participants predominantly appraised demands as a threat. A small number of demands were appraised as a challenge or benefit, and the fewest demands were appraised as causing harm or loss. Participants’ appraisals were informed by underlying properties of stress appraisal such as self and other comparison, and preparation. Participants often relied on personal resources as opposed to available workplace resources. They perceived well-being to relate to stress appraisals with participants experiencing acute and long-term outcomes.

**Discussion:**

This study offers insight into the lived experience of the occupational stress process within professional classical musicians. The findings demonstrate that organizational interventions targeted at continuing professional development and social support are appropriate to help musicians cope more effectively with demands.

## Introduction

Professional classical musicians experience a variety of demands due to the occupational environment within which they operate. This includes intrapersonal, interpersonal, and organizational demands (Willis et al., 2019). On an individual level, classical musicians are required to attain a high level of technical proficiency whilst also demonstrating emotional expressivity during performance (Williamon, 2004). Interpersonal demands arise due to relational issues with colleagues and management. In addition, freelance musicians require business acumen and self-promotion skills when engaging with key stakeholders to ensure further work opportunities (Kubacki, 2008; Coulson, 2012). Ensemble musicians also report a high demand on their interpersonal skills, which are needed to facilitate musical communication and positive relationships with colleagues (Lim, 2014). At an organizational level, musicians experience demands due to scheduling, touring, low remuneration, and contract insecurity (Rickert et al., 2013; Vervainioti and Alexopoulos, 2015). In the UK, a large number of orchestral musicians work in a freelance capacity (Association of British Orchestras, 2019) meaning that these individuals receive short-term contracts with irregular work patterns. If not coped with effectively, the demands faced by professional classical musicians can cause occupational stress and threats to well-being (Willis et al., 2019).

Professional classical musicians draw on internal and external resources to cope with the demands experienced. Musicians value the importance of teamwork, a feeling of community amongst colleagues, and collaborative working environments (Brodsky, 2006; Ascenso et al., 2017). Researchers have demonstrated, for example, that social support from colleagues can help manage the stress response resulting from career insecurity and emotional demands (Parker et al., 2019; Pihl-Thingvad et al., 2022). Musicians have also described active coping skills such as planning, problem solving, consulting medical professionals, learning to handle disappointments, and maintaining good physical health in order to protect their well-being against the occupational demands experienced (Burland and Davidson, 2002; Sandgren, 2002; Pecen et al., 2018).

Both demands and resources may affect professional classical musicians’ well-being outcomes. The intra-individual process(es) whereby they do this are not well understood. Willis et al. (2019) asserted that holistic approaches to assessing occupational stress and well-being in musicians should be taken. One approach is by using a Cognitive-Motivational-Relational Theoretical approach (CMRT) (Lazarus, 1999). This approach may provide insight into the stress processes experienced by an individual musician, particularly in why and how they appraise occupational demands.

In his CMRT, Lazarus (1999) suggested that stress should be viewed as a transaction, incorporating the ongoing relationship between the individual and the environmental demands they encounter, with emotional and well-being outcomes resulting from such transactions. Lazarus (1999) argued that the demands experienced may arise from a variety of contexts such as the workplace, family, or wider cultural expectations. Central to the experience of stress in the face of such demands is the concept of appraisal. Split into primary and secondary appraisals, Lazarus suggested that for an individual to view a demand as stressful, the demand must first be appraised to be relevant to their personal goals or motivations. Such demands may be appraised as threatening, a positive challenge (i.e., an opportunity for growth), beneficial, or harmful (primary appraisal). An additional layer to primary appraisals is the concept of underlying properties. Specifically, Lazarus and Folkman (1984) suggested that at least one of eight properties is required for an individual to make a stress appraisal. These include novelty, predictability, event uncertainty, imminence, duration, temporal uncertainty, ambiguity, and the timing of stressful events in relation to the life cycle (Lazarus and Folkman, 1984). Thatcher and Day (2008) extended this work and examined the underlying properties of stress appraisal in a sports context. Based on their findings, they expanded the factors of underlying stress appraisal to include “self and other comparison” and “inadequate preparation.” Definitions for each underlying property of stress appraisal are provided in Table 1 alongside an example of how this could translate to a musical context. Secondary appraisal then involved the individual evaluating whether they have the resources available to cope with the demand, which Lazarus (1999) differentiated from the act of coping.

**Table 1:** Definitions of underlying properties of stress appraisal (Lazarus and Folkman, 1984; adapted from Thatcher and Day, 2008).

Underlying property of stress appraisal	Definition	Example
Novelty	Situations that the person has not previously experienced. Previous experience may include both experiencing a similar situation and information that can be read, heard or inferred	First orchestral trial
Predictability	When established expectancies are no longer met the situation becomes unpredictable	A change in rehearsal or performance schedule compared to usual
Event uncertainty	The likelihood or probability of an event’s occurrence. These can be subjective or objective probabilities although subjective estimates do not necessarily match objective ones	Subjective probability: The likelihood of performing correctly Objective probability: The likelihood of a performance taking place
Imminence	The period of anticipation before an event occurs	Anticipation whilst travelling to a performance
Duration	The length of an event. Events of a long duration will be deemed more stressful than those of a short duration	Tours taking place over several months
Temporal uncertainty	The individual knows that an event will definitely happen but is unsure of the precise timing	Waiting to be called into an audition
Ambiguity	When the information needed for appraisal is unclear or insufficient resulting from a lack of situational clarity	An unknown conductor leading a performance
Timing of events in relation to life cycle	Events occurring at the same time as other stressful events in the individual’s life cycle may be appraised in relation to these other events	Performances taking place during a period of increased caring responsibilities
Self and other comparison	Comparing any physiological, psychological, or social aspect of performance or the associated environment with that of another individual	Comparing personal performance of a piece of music to a colleague’s performance
Inadequate preparation	The individual does not feel well prepared for performance	Poor practice prior to a performance

Dependent on the results of individuals’ appraisals of their coping resources, they may attempt to use cognitive, emotional, or behavioral coping strategies (Lazarus, 1999). Lazarus and Folkman (1984, p. 141) argued that coping is a process and defined it as “constantly changing cognitive or behavioral efforts to manage specific external and/or internal demands that are appraised as taxing or exceeding the resources of the person.” In other words, coping is the steps or actions an individual uses to manage the demands they experience. Lazarus (1999) suggested two distinct factors of coping: problem-focused and emotion-focused. Problem-focused coping refers to taking action and using strategies that change the relationship between the person and the environment, (e.g., planning, problem solving). Emotion-focused coping describes efforts to regulate an emotional response through strategies such as venting about a situation or avoiding a demand. Research on the resources individuals use to cope with demands has been extended by the JD-R theory, which includes occupational resources and personal resources (Bakker and Demerouti, 2014). Schaufeli and Bakker (2004) suggested that occupational resources can decrease occupational demands, support the achievement of work goals, and contribute to personal development and well-being.

Considering conceptualizations of well-being, both hedonic and eudaimonic well-being are relevant to the occupational experiences of musicians (Willis et al., 2019). Hedonic well-being is made up of affective and cognitive dimensions (Diener et al., 1999). The affective dimension of hedonic well-being includes positive and negative affect, and the cognitive dimension is represented by perceived satisfaction. Eudaimonic well-being is more holistic and includes six dimensions: self-acceptance, positive relations with others, autonomy, environmental mastery, purpose in life, and personal growth (Ryff, 2014). Bartels et al. (2019) argued that hedonic and eudaimonic well-being are distinct despite being highly correlated. Therefore, hedonic and eudaimonic well-being can be considered complementary (Waterman, 2008; Huta and Waterman, 2014), and VanderWeele et al. (2020) suggested that studies of psychological well-being examine both concepts.

Through CMRT, Lazarus (1999) argued that appraisals will influence the emotions experienced. For example, threat may cause anxiousness and challenge may lead to excitement. Such emotional experiences may be conceptualized as the affective dimension of hedonic well-being. Additionally, the reciprocal nature of experiences of stress and emotion, respectively, as depicted in CMRT, can give rise to longer-term well-being responses such as satisfaction from the knowledge of coping well with stressful encounters.

In the literature on classical musicians, no study has considered a holistic approach to understanding stress and well-being, which incorporates demands, appraisals, coping, and well-being. A holistic approach that is underpinned by CMRT has been adopted in other demanding performance environments such as sports to explore the occupational stress process and well-being outcomes (e.g., Neil et al., 2016). Such studies have examined appraisals, underlying properties of appraisals, and coping strategies and their effectiveness (e.g., Hanton et al., 2012; Didymus and Fletcher, 2014; Didymus, 2017). Notably, an individual’s experience of the stress process has been linked to well-being outcomes (Neil et al., 2016).

Like athletes, musicians work in situations with high performance demands. As such, the potential exists for comparable experiences of occupational stress and well-being. However, as identified by Willis et al. (2019), no qualitative studies underpinned by CMRT have yet considered the stress process and well-being outcomes in classical musicians. Given that this theory, with the inclusion of appraisal and associated underlying properties of stress appraisal, has successfully been used to inform approaches for exploring stress and well-being outcomes in high performance-based roles, this study explored these concepts in professional classical musicians.

### Aim and research questions

The aim of this study was to interpret the lived experiences of perceived occupational stress and well-being of professional classical musicians through a rich understanding of the demands they face, the appraisals they make, the resources they use, and their perceived influence on self-reported well-being. The following research questions framed the research design:

*RQ1*: What are the perceived demands associated with the lived experiences of professional classical musicians?

*RQ2*: What primary appraisals do professional classical musicians report when experiencing occupational demands?

*RQ3*: What occupational and personal resources do professional classical musicians report using to cope with the occupational demands experienced?

*RQ4*: How is well-being experienced by professional classical musicians when encountering occupational demands?

*RQ5*: What are the perceived connections between occupational demands, primary appraisal, occupational resources, personal resources, and perceived well-being outcomes?

## Methods

This study is reported in accordance with the journal article reporting standards for qualitative research (Levitt et al., 2018).

### Philosophical position

This study adopted a critical realist position (Bhaskar, 2008), in which an independent reality exists “out there” (O'Mahoney and Vincent, 2014). The authors of the present study acknowledge subjectivity in how individuals perceive reality and reject the notion of multiple realities (Fleetwood, 2014). Aligned to this philosophical position, the study used Interpretative Phenomenological Analysis (IPA) (Smith et al., 2022; Smith and Nizza, 2022).

### Research design

The focus of an IPA design is the deep exploration of the lived experiences of participants. IPA considers participants’ experiences alongside their actions, thoughts, and feelings (Smith and Nizza, 2022). The theory of IPA is drawn from the areas of phenomenology, hermeneutics, and idiography. IPA is interpretative on two levels: first, the participant interprets and makes sense of their own experiences and second, the researchers are involved in interpreting the participants’ sense-making (Smith and Shinebourne, 2012). This process is referred to as a “double hermeneutic” (Smith and Osborn, 2003). The interpretative function of the researchers is central to IPA, although it is acknowledged that the phenomena is viewed primarily through the rich descriptions and details provided by the participant (Smith et al., 2022).

IPA was adopted for this study as a suitable means to explore the phenomenon of occupational stress as it relates to associated thoughts (i.e., primary appraisal, cognitive well-being outcomes), actions (i.e., use of personal and occupational resources), and feelings (i.e., affective well-being outcomes). The idiographic approach of IPA was considered appropriate to explore both appraisals and perceived well-being as these are subjective. Further, appraisals are often made using unconscious schema, meaning it can be difficult to gain access to these cognitive processes using nomothetic approaches. An idiographic approach involves considered questioning and allows individuals adequate time for reflection in order to provide detailed accounts of their personal experiences regarding their thoughts and feelings when encountering occupational demands.

### Researcher characteristics and reflexivity

To interpret the participants’ lived experiences, the primary researcher engages closely with the interview transcripts. This requires the researcher to put aside or “bracket off” their initial ideas and preconceptions about a topic (Pietkiewicz and Smith, 2012; Smith et al., 2022). This bracketing off is not only necessary prior to conducting interviews but is a continual process throughout the research (Smith et al., 2022). In this study, the primary author engaged with reflexive interviews with research team members, framed by the reflexive interview model developed by Dallos and Vetere (2005).

This process necessitates some key positionality acknowledgements. The first author completed an undergraduate music degree at a UK Conservatoire and previously worked as a musician. This work mostly involved peripatetic teaching and included a small number of freelance performances within orchestral and chamber music settings. In this way, the first author was familiar with the professional classical musicians’ practice community. The last author was previously involved in a wide variety of research on classical musicians’ health and well-being. The second and third authors are experienced researchers and practitioner psychologists with extensive experience of working in mental health, well-being, and performance psychology across a variety of demanding performance driven environments. They had not previously worked in classical music.

To encourage reflexivity during the study, a research journal was kept throughout the process of data collection and analysis. Journal entries included initial thoughts on the process of interviewing, thoughts and feelings about interviews in relation to prior experience, progress notes, conceptual notes, and ideas relating to existing literature. Regular meetings were held between the research team throughout the data analysis process to discuss and critically challenge the development and refinement of themes.

### Sampling, recruitment, and participants

In this study, participants were recruited from a previously utilized research population (Willis et al., in preparation). To be eligible to participate, musicians needed to be professional classical musicians, defined as individuals earning the majority of their salary through performance and music-related activities (e.g., teaching). All instrumental and vocal categories were eligible and participants were required to be aged 18 or above. Musicians were excluded if they were amateur musicians or mostly performed music of a different genre (e.g., popular music). Musicians who had not completed the full questionnaire in the previous study were not eligible to participate in this study.

Given that the previous questionnaire study had included questions regarding demands, stress, resources, and well-being, it was perceived that these individuals had the experiences necessary and motivation to be potential participants for the present study. Their participation would better ensure the homogeneity of participants as per IPA expectations (Smith et al., 2022). To reflect the idiographic approach of IPA, a small sample was recruited (*n* = 6).

Recruitment was carried out between January and February 2021. Participants from the questionnaire study were contacted via email and invited to participate. Of those who volunteered, 16 professional musicians were eligible to participate. Five volunteers from outside the UK were excluded to assure participant homogeneity. Six professional musicians were contacted and all agreed to be interviewed. Participant demographics are presented in Table 2. Participants consisted of two full-time contracted musicians and four were self-employed. Participants included four males and two females and ranged in age between 29 and 54. Two participants played string instruments, one of whom was also a conductor, two played woodwind instruments, and two played brass instruments.

**Table 2:** Participant demographics.

Name	Gender	Age	Instrument	Employment
Adam	Male	43	Woodwind	Employed
Ben	Male	29	Brass	Self-employed
Charlotte	Female	48	Woodwind	Self-employed
Daniel	Male	52	Brass	Employed
Eva	Female	49	Strings	Self-employed
Kieran	Male	54	Conductor/Strings	Self-employed

### Instrumentation

An interview schedule was developed and included questions that would allow participants to provide a “rich” account of their experiences. Questions were designed to be open-ended and focused on the types of occupational demands experienced, appraisal of those demands, resources, and perceived well-being outcomes (see Supplementary material). Participants were asked to provide examples of specific situations in which they had encountered an occupational demand and explored their experiences, actions, thoughts, and feelings. Participants were asked to reflect on two stressful events: one they perceived as a positive experience and one which was a negative experience. Given that interviews took place during the COVID-19 pandemic, some participants were working under exceptional circumstances or had minimal employment within the classical music sector at the time of interview. To enhance the relevance of the research to the broader occupational experiences of classical musicians, participants were asked to discuss events that had occurred prior to COVID-19 or those that were minimally impacted by COVID-19. The interview schedule was piloted with two musicians from the professional network of the first author. Pilot interviews took place between October–December 2020 and included musicians with a range of occupational experiences (including freelance, permanent employment, and chamber music work). Following the pilot interviews, minor changes were made to the interview schedule to refine the clarity of questions, use of prompts, and usability of the interview guide.

### Procedure

Ethical approval for this study was provided by the University Ethics Committee. Prior to interview, participants were provided with information sheets and completed a consent form. Participants were reminded of their voluntary involvement and right to withdraw. As a safeguarding consideration, participants were asked to use a location away from others to help safeguard the anonymity of anyone discussed during the interview and maintain confidentiality of the interview content. After completion of the interview, participants were signposted to appropriate organizations should they experience a significant reaction to the interview resulting in a high level of stress and impact on well-being.

Six semi-structured interviews lasting 63–126 min (mean = 92 min) were conducted by the first author with individual participants in February 2021. Due to the COVID-19 pandemic and the necessity to maintain social distancing measures laid out by the Welsh and UK Governments, all interviews were conducted via an online video conference platform (Zoom; https://zoom.us/). Interviews were audio recorded using a digital audio recorder and notes were made following interviews. Interviews were transcribed using an automatic online transcription software, Transcribe.^1^ Transcripts were then reviewed, manually edited, and pseudonymised. Participants were given the opportunity to check and reflect on their transcript to ensure that text and pseudonyms provided sufficient confidentiality and that they were in agreement that the narrative account was a “genuine” and “authentic” representation of the interview (i.e., member check). Some participants made minor clarifications about the meaning of phrases at this stage.

### Data analysis

Data analysis took place between September 2021 and September 2022, and IPA protocols were followed throughout (Smith et al., 2009; Smith and Nizza, 2022). Transcripts were imported into NVivo 1.3 (2020). Transcripts were read and reread by the first author for familiarity and initial exploratory notes were made. Exploratory notes included descriptions of participants’ experiences, comments on the use of language, and conceptual notes. From these exploratory notes, experiential statements were developed. Experiential statements were generated from components within CMRT, related psychological concepts, and concise phrases that reflected the exploratory notes made on the transcript. Experiential statements were then clustered into groups through abstraction and subsumption to create Personal Experiential Themes using MindView 7 (MatchWare, 2017, see Supplementary Figure S1). Following this, Personal Experiential Themes were contextualized according to conceptual and temporal elements of the participants’ experiences to visually reduce the data. Memos were written detailing how experiential statements fitted within Personal Experiential Themes and exploring relationships between superordinate themes.

Following this, Personal Experiential Themes across transcripts were compared. This was done by clustering Personal Experiential Themes into Group Experiential Themes using mind maps and comparing memos across participants. A table of Group Experiential Themes was created for cross-case analysis and used to refine themes at the group level (Smith and Nizza, 2022). This table included quotes and descriptions of each participant’s experience of the stress process. Group Experiential Themes related to the different types of demands that participants experienced.

To address the research questions, participants’ experience of each demand was considered in turn, in accordance with elements of the stress process. As such, the table of Group Experiential Themes was expanded to include primary appraisals, underlying properties of stress appraisal, resources, and well-being aligned to each demand described. Each was categorized, described, and an example quote was noted. Categorization was consistent with concepts in CMRT (Lazarus, 1999; Thatcher and Day, 2008), and hedonic and eudaimonic well-being (Diener et al., 1999; Ryff, 2014). Appraisals were categorized as either threat, challenge, benefit, harm, or loss; underlying properties of stress appraisal were categorized as one of the 10 dimensions identified by Thatcher and Day (2008); resources were categorized as either personal resources or occupational resources; perceived well-being experiences were categorized as either hedonic (positive affect, negative affect, or satisfaction) or eudaimonic (autonomy, environmental mastery, personal growth, positive relations with others, purpose in life, or self-acceptance).

As the initial development of Personal Experiential Themes and Group Experiential Themes was conducted by the first author, critical reflective and reflexive discussions were held with co-authors throughout the data analysis process. Within these meetings, the categorization of experiential statements into Personal Experiential Themes was challenged, preconceived conceptual ideas were discussed, and the influence of insider knowledge was critiqued to better assure interpretations were trustworthy. Additionally, the conceptual representation of superordinate themes and the mind maps created for each participant were critiqued. Further, the conceptual framing of Group Experiential Themes and associated participant quotes was critiqued.

## Results

In this section, participants’ experiences of the occupational stress process and perceived well-being outcomes are detailed and illustrated by quotations. All six participants are represented in the quotations chosen. Through analyzing the transcripts, three Group Experiential Themes were developed, which were encompassed under Occupational Demands: (a) Performance Demands; (b) Organizational Demands; (c) Relationship Demands. Within each Group Experiential Theme, examples of specific demands, primary appraisal, underlying properties of stress appraisal, resources, and perceived well-being outcomes are illustrated. These components of the stress process are presented together to illustrate the depth of participants’ experiences and to preserve the idiographic quality of IPA. Through this representation, the research questions are clearly addressed. A summary of Group Experiential Themes and the types of appraisal participants described is provided in Table 3. An overview of all participant experiences is presented in the Supplementary material.

**Table 3:** Summary of group experiential themes.

Group experiential theme		Example participant experiences	Type of appraisal				
			Threat	Challenge	Benefit	Harm	Loss
1.	Performance Demands	Daniel: Chamber music performance Eva: Recording project	23	5	7	1	–
1.1.	Performance Standards	Daniel: Maintaining performance standards Ben: High performance standards on tour	6	1	–	–	–
2.	Organizational Demands	Kieran: Touring	21	3	3	–	–
2.1.	Role Demands	Adam: Multiple roles Adam: Presenting concert	4	1	1	–	–
2.1.1.	Responsibility	Eva: Orchestral leader	4	–	–	–	–
3.	Relationship Demands	Charlotte: Colleagues’ argument Adam: Management staff relationship	7	–	3	2	2

### Theme A: Performance Demands

Performance Demands are operationalized as those demands that related to participants’ experience of the musical demands they encountered (e.g., technical demands, musical interpretation demands) and demands directly arising from performance contexts (e.g., recording demands). All participants discussed Performance Demands. Participants largely appraised Performance Demands as a threat (23 appraisals), followed by appraisals of benefit (7 appraisals), challenge (5 appraisals), and harm (1 appraisal).

Daniel was employed as an orchestral musician and occasionally performed chamber music. Daniel discussed performance *demands,* describing the need for greater stamina when performing chamber music due to the need to play more continuously in comparison with orchestral repertoire. Daniel used the simile of a sprinter to describe the differences:

*It’s a bit like asking a sprinter to do a 10 k race… You would not say, “Okay. We’re just been marking you on your sprinting… Go and do a 10 k and be back in forty-five minutes.” … it’s a similar situation of brass players doing chamber music. So it’s different.*

Daniel *appraised* performing chamber music as threatening, demonstrated in his feelings of discomfort: “Like a fish out of water. I feel like a fish out of water.” However, Daniel had previously benefitted from performing chamber music and saw the potential to benefit again: “I think, is really good for us… if it’s handled correctly.” Daniel described the *underlying properties of threat appraisal* of preparation and self and other comparison. Daniel perceived that there was insufficient rehearsal time to achieve a high level of artistic quality: “I think we need preparation… we should be given more… We should be given extra time.” Considering self and other comparison, Daniel compared the standard of chamber music he could achieve currently with his past experience:

*Having done it to a high level in the past but quite a long time ago, I’ve got that standard of chamber music in my head and it’s very difficult to replicate that just by throwing a bunch of orchestral musicians together and giving them a day or two.*

Regarding “relaxed” performances, Daniel discussed the work resources of relatedness and autonomy: “it’s a very collegiate atmosphere in the rehearsals” and “it’s quite a musician led project… That works quite well.” However, Daniel perceived a lack of organizational *resources* such as time and artistic management: “that’s not really being managed artistically at all [laughs].” Despite his discomfort, Daniel experienced a positive affective *well-being* outcome when performing in some chamber music contexts, particularly relaxed concerts. However, overall, Daniel experienced a lack of satisfaction from chamber music performances as he did not find them as beneficial as they could be: “I think are not, are not as satisfying as they could be. Not as satisfying as they should be.”

Considering the performance *demands* of a recording project, Eva discussed rehearsing and recording a large amount of material within a tight timeframe: “there is then pressure… if you did not get it right, there is no time to get it right… You have to get it right now [laughs].” Eva needed to perform at a high standard as she was the soloist: “trying to do that absolutely perfectly… a kind of almost unrealistic quest for perfection.” Eva *appraised* the recording as a challenge and opportunity for development: “a very nice… way of… growing out of the leader’s chair into the soloist chair.” Reflecting, Eva appraised a benefit in terms of industry esteem recognition and financial reward. In terms of the *underlying properties of challenge appraisal*, Eva discussed duration and preparation: “I made an arrangement… I prepared that. I prepared the arrangement and the put the music…” Eva used multiple work *resources* including social support and autonomy. Concerning her colleagues, Eva reported tangible support from a producer who took responsibility for scheduling: “they keep an eye on the time… and it was a producer I trusted with my life.” Eva discussed emotional support from the orchestral musicians: “it was a wonderful… feeling of being supported… almost carried, carried in a nice way [laughs],” as well as feelings of relatedness. Eva also experienced autonomy demonstrated through the repetition of the word ‘I’: “I chose the repertoire and I made an arrangement of a harpsichord concerto… I prepared that. I did that. I arranged that. I prepared the arrangement.” Eva reported a number of *well-being* outcomes related to this project. Considering positive affect, Eva experienced excitement during the recording due to the appraisal of challenge, and gratitude towards her colleagues. Regarding the finished recording and the appraisal of benefit, Eva was proud: “I am very proud of it. Of course, I’m proud of it… So [laughs] so, so that’s brilliant.” Eva also discussed eudaimonic well-being and the dimensions of environmental mastery, positive relations with others, and personal growth. Eva demonstrated environmental mastery in her contribution to the artistic direction of the project and through creating arrangements. Eva also developed her practice as a soloist and reported personal growth linked to the benefit appraisal.

Within the theme of Performance Demands, participants discussed demands created by the performance standards required for their roles. Participants described the need to perform to a high standard, which was closely linked with their own and others’ expectations. Performance standards were discussed by three participants: Adam, Ben, and Daniel. Participants mostly appraised performing at a high standard as a threat (6 appraisals), followed by challenge (1 appraisal).

Daniel discussed the *demands* of maintaining high performance standards: “recently the day-to-day demands are, for me, are meeting my own expectations.” Daniel discussed an occasion when he had done some freelance work outside his employed role. During this, Daniel felt he was not performing at his usual high standards, a feeling that continued when he returned to his substantive role: “I felt as though my playing had gone off track a bit… gone off course a little bit.” Daniel *appraised* the situation as threatening due to the possibility of being judged unfavorably by his colleagues: “trying to make sure that… the people around me do not notice a deterioration in my playing [laughs].” Daniel experienced multiple *underlying properties of threat appraisal*: preparation, event uncertainty, and self and other comparison. The performance demands were in the area where Daniel was struggling and he felt unprepared. As a result, Daniel experienced event uncertainty in the form of subjective probability and was unsure whether he could perform at the requisite standard. Further, Daniel compared his performance standards now with his performance standards across his career:

*When you start off on an instrument your, your progress curves. Your progress line is very steep… once your playing’s plateaued and you think, oh, hang on… I’m still putting the work into my playing but it’s not getting any better… this is something that I’ve always taken for granted in my playing and suddenly it feels a bit difficult… you start thinking about that trajectory and you almost, you almost feel like you are on a ballistic trajectory where you have gone up and now you are going down.*

Daniel discussed the work *resource* of colleague social support. On the one hand, Daniel perceived his colleagues as emotionally supportive: “if somebody’s genuinely struggling then, then people have a lot of sympathy.” On the other hand, Daniel perceived a lack of emotional support from some colleagues: “there are people who, they will not have as much time for somebody who’s not, who’s struggling with their playing.” Daniel also discussed the personal resources of routine practice and mindfulness. Considering mindfulness, Daniel focused on the present moment: “focusing back on the breathing and… the music.” Regarding hedonic *well-being*, Daniel experienced negative affect due to the appraisal of threat: “I felt very uncomfortable.” Daniel also reported a negative impact on eudaimonic well-being in terms of environmental mastery and his concern about losing skills when he discussed being on a “ballistic trajectory.” However, during the performance, Daniel’s use of mindfulness restored his feelings of mastery and he was able to perform to a high standard.

Ben also discussed *demands* related to performance standards, which he experienced whilst on an international tour. Ben had been asked to join the tour at very short notice. Although he had not attended the main rehearsals, he needed to fit in with the orchestra members, who were familiar with the repertoire. As a freelancer, Ben understood that being “able to read music quickly and react to it” was a key part of his role. Ben *appraised* the situation as a challenge and believed he would perform “to a good enough standard, if not a high standard.” Ben experienced the *underlying property of challenge appraisal* event uncertainty in terms of his ability to perform at the required standard, feeling “quite confident” in his abilities. Ben discussed using personal *resources* in the form of personal practice and emotion regulation. Ben described how emotion regulation helped him to stay in control of his performance:

*Try and temper the excitement and the, the energy and the—hate the word but—passion and the emotion… try and put it to one side and just focus on what you are doing, but still be aware that you do enjoy it.*

Ben’s feelings of confidence reflect the environmental mastery element of eudaimonic *well-being*. Having performed successfully on the tour, Ben reported an increased perception of mastery, which implied that he appraised the experience as a benefit on reflection.

### Theme B: Organizational Demands

Organizational Demands are defined as those demands controlled at the organizational level (i.e., by management staff). These demands included travel, scheduling, and role related demands. Organizational Demands were discussed by all participants and were predominantly appraised as a threat (21 appraisals), followed by benefit (3 appraisals), and challenge (3 appraisals).

Kieran discussed organizational *demands* related to a world tour in terms of accepting the offer to tour and the schedule. He discussed needing to make a quick decision whilst also considering his family:

*They said, “Look, we are off on a world tour next year… would you come and conduct all of the orchestras?” To which this time, I did not consult with my wife. I just said, “Yes.” And then went home and we had a consultation.*

Kieran *appraised* a threat, in that he might lose the opportunity if he did not act quickly: “if you say no, people will just move on.” Kieran also appraised the tour as a challenge: “It was an opportunity came and, and I thought, right. I’m going to try this. I’m going to go headlong into it.” In this situation, Kieran experienced the *underlying property of threat appraisal* event uncertainty as he was unsure how long the opportunity would be available. He accepted the opportunity with the knowledge that he could later refuse. Kieran discussed the personal *resource* of family social support: “we sat down and we made that choice as a group, as a family… So those things are always important.”

Considering the tour itself, Kieran discussed the *demands* of travelling and performing in different countries: “You’d be in Taiwan for three or 4 days, and then you were in China, and then Indonesia, then Malaysia, then to Japan, then Hong Kong…” Kieran *appraised* the tour as a challenge and on reflection made an appraisal of benefit: “I learned much about myself by doing the touring.” He later said, “[Touring] gave me what I have now… career as a conductor and as a, as an all-round portfolio-type musician.” Kieran discussed the *underlying property of benefit appraisal* of novelty in relation to visiting different countries: “there were always new experience to have, new foods to try.” Kieran thought of the tour as a development opportunity, which is a work *resource*: “I gave myself the opportunity to take that and run with it.” Kieran discussed several positive *well-being* outcomes. Related to the appraisal of challenge, Kieran experienced positive affect: “that was terribly exciting.” Regarding the appraisal of benefit, Kieran experienced the positive affective outcome of gratitude. Further, linked to the appraisal of benefit, Kieran experienced the eudaimonic well-being outcome personal growth by realizing what was important:

*I learned that I did not need quite so much stuff in my life… there were certain things that were very important to me… two little books, one, one for each tour, my wife built for me with pictures of my kids in… I had little notes that were written by the children… So, those things were important. But I realized that I did not need, you know, oodles and oodles and oodles of clothes.*

Additionally, Kieran experienced increased environmental mastery through conducting on the tour: “really opened my eyes to so many other things. Of what I was, I thought that I probably wasn’t capable of… And that I became very capable of eventually.”

#### Role Demands

Within Organizational Demands, participants discussed role-related demands, such as role insecurity, role conflict, and role strain. Role conflict related to the demands of holding multiple roles within and across organizations, and role strain related to the level of responsibility a musician had within their organization. Role Demands were considered by Adam, Ben, and Daniel. Mostly, participants appraised Role Demands as a threat (4 appraisals), followed by challenge (1 appraisal), and benefit (1 appraisal).

Adam discussed role conflict and the *demands* of performing multiple roles at the same organization. Due to a vacancy, Adam was required to perform in his employed role and two additional roles. Although the roles were related, they required changes to Adam’s performance: “that’s probably one of the biggest demands for me… I like to think of it as wearing different hats… even moving two seats along left or right, it’s amazing how different it feels.” Adam discussed a chamber music performance where he played in the principal position. Adam *appraised* a threat in this situation and experienced role instability: “there’s a sort of fragility to it…” Further Adam appraised a threat due to the potential for being judged negatively and being asked not to perform in the principal role: “The management have the power to say, ‘I’d rather you did not do that anymore.’” Adam experienced the *underlying property of threat appraisal* temporal uncertainty when performing in the principal role as he knew the role was being advertised yet he did not know when he would be required to stop performing the role. Considering *resources*, Adam used the psychological technique of minimization: “I’m quite happy where I am… I have not got much ambition to sort of climb the ladder and become a principal.” Adam implied that this experience had a negative impact on his *well-being* due to the appraisal of threat and he discussed the issue with management: “I felt I needed to communicate what though, what that… for my own confidence and my own mental health.”

Adam discussed another occasion when he experienced role related *demands* due to presenting a concert: “there’s been recently opportunities to stand up front, in front of the orchestra and be a presenter.” Adam introduced the concert, talked to the audience between pieces, and performed his instrumental role. Adam *appraised* the demands as challenging: “Yeah, I also had to play. That was… also the challenge.” Further, Adam appraised a benefit to his career from presenting: “something that stands me out from my other musicians.” Adam experienced the *underlying property of challenge appraisal* of novelty as this was the first time he had presented a concert for the full orchestra. Adam also experienced self and other comparison as an *underlying property of benefit appraisal*:

*Here’s something I think I can do that not a lot of my colleagues can do. Because a lot of my colleagues would not ever stand up and talk to… a big audience. And a lot of them to be honest probably would not be very good at it.*

Adam drew on the same personal *resources* he used for musical performances and used imagery during rehearsal: “imagining that there was the audience there… it’s exactly the same thing that I have done… as a musician.” Adam described his preparation: “I scripted what… I was going to say and then I condensed it into bullet points, and then I put those bullet points… onto a card.” There was a positive impact on Adam’s hedonic *well-being* as a result of his appraisals of challenge and benefit: “I felt very good. I felt very good doing it actually, which is probably why I want to do more of it because it felt like a, a good experience… I felt very happy.”

##### Responsibility

Role Demands also encompassed role strain and the level of responsibility participants held. Demands relating to responsibility were discussed by Charlotte, Eva, and Kieran, and were solely appraised as a threat (4 appraisals).

Considering a prestigious orchestral performance, Eva had an elevated level of responsibility: “I was guest leading it, I usually just play in the section there.” Eva discussed the responsibility of an orchestra leader and the *demands* this created:

*You have to make sure that the conductor gets what, what he or she wants to get and then the orchestra’s needs are met. As in, is the conductor clear enough? … you have to play and you have to lead your section, so that your body language… conveys how and when the section should play… And if there’s any question, if the conductor is not clear at any point, then the leader’s job is to kind of rescue the situation and be very clear and, and save the day, as it were. And gave a big gesture—here, we are now.*

Within this scenario, the conductor made an unexpected and unclear gesture: “In the concert X Conductor did something… slightly different from what… [they] did in every rehearsal.” As the leader, Eva felt it was her responsibility to “rescue the situation… and save the day.” However, Eva was not able to bring the performers together: “there was a bit of a car crash.” Eva *appraised* a threat to her employment as she had not adequately fulfilled the role of leader: “being in the, in the leading chair, there is a responsibility with you.” Further, she appraised a threat to her employment due to her colleagues’ perception of the event: “It made me feel fearful of what my colleagues think of me.” Eva also appraised the event as causing lasting harm: “it leaves you pretty, you know, pretty… scarred.” Eva reported the *underlying property of threat appraisal* of self and other comparison in two ways: firstly, there is the implication that Eva compared her leadership to the ideal she described; secondly, Eva was concerned about being negatively evaluated by her colleagues according to acceptable industry standards. Additionally, Eva experienced ambiguity in trying to evaluate whether she had correctly interpretated the conductor’s gesture: “whether I was right or wrong, I still do not know.” To cope, Eva used workplace *resources* and sought emotional and informational social support from her colleagues. Eva used informational support to address the ambiguity: “I wanted an honest opinion. What they think happened… That was a good way of… getting some clarity for myself.” Additionally, Eva used psychological skills in the form of self-talk to regain a sense of control:

*It left me needing to have a kind of chat with myself and think, okay. So, what exactly happened? What could I have done differently? … what aspect of that was, you know, something that was in, that is in my control to change now?*

This experience had a lasting negative hedonic *well-being* outcome related to Eva’s appraisal of harm and she experienced anxiety: “even now when I talk about it… I feel the knot in my stomach.” Eva was also dissatisfied and felt disappointment: “It made me… disappointed with myself.” Reflecting, Eva moved towards self-acceptance, a dimension of eudaimonic *well-being*: “I wasn’t sure that in that moment I could have done anything differently… so, like an instinct, instinctive reaction.”

### Theme C: Relationship Demands

Relationship Demands are defined as demands that involved interpersonal relationships between musicians and their colleagues and/or management staff. Four participants discussed Relationship Demands: Adam, Ben, Charlotte, and Kieran. Relationship Demands were appraised mostly as a threat (7 appraisals), followed by appraisals of benefit (3 appraisals), harm (2 appraisals), and loss (2 appraisals).

Charlotte discussed an occasion when an argument had taken place and created a relationship *demand*: “During the rehearsal… [there] was an enormous row between… a small group of players, including my co-soloist and the management… about the way the stage was set.” Following the argument, one of the musicians left the rehearsal. This occasion related to a performance where Charlotte was performing as a co-soloist, which was a role she performed infrequently. Charlotte made an *appraisal* of threat and perceived a risk of becoming involved in the argument:

*Initially, I was not in the same room… I’ll just keep my head down and stay out the way. Because the last thing you want when that’s going on is to stick your head round the door and find out.*

After the concert, Charlotte appraised that loss and harm had been caused. Considering loss, Charlotte said of the colleague who left the rehearsal, “You spoiled a day that was a really important one for me.” Considering harm, Charlotte used the simile “it just felt like we’d all had a kicking.” Although not part of the argument, Charlotte was vicariously affected and experienced the *underlying property of threat appraisal* of predictability. This was due to the fact that usual workplace norms regarding colleague interactions had not been followed. Considering the underlying property of the loss appraisal, Charlotte experienced novelty as this was not her typical experience. Charlotte employed personal *resources* and used avoidance and emotion regulation. Regarding avoidance, at the time of the argument Charlotte tried to “stay out the way,” and additionally avoided the situation during the break. Charlotte also focused on her breathing: “I try and remember to breathe [laughs]—really deeply… if you concentrate on it, you can help yourself a lot.” Charlotte discussed multiple negative hedonic *well-being* outcomes. Related to the appraisal of threat, Charlotte experienced anxiety: “unsettling, upsetting, unpleasant.” Linked to the appraisals of harm and loss, Charlotte experienced anger and sadness: “I just felt really angry,” and “it just left me feeling incredibly upset.” Further linked to the harm and loss appraisals, Charlotte was dissatisfied as the experience had not lived up to her expectations: “I felt upset because usually I get a lot of positivity from engaging in ensemble work, especially with that group of people.” Regarding eudaimonic well-being, the dimension positive relations with others was negatively affected, particularly with the musician who left the rehearsal: “I just thought you selfish so-and-so.”

Adam described how his relationship with management staff created a *demand* for a chamber music performance:

*[Pause] Now [Pause] I want, you see [laughs] there was, there is a bit of background to this in that prior to that… there was a, a miscommunication from my management, where they said… that they were going to get… a guest in to play that part, rather than me, have me do it… So, the reason for this was because, as I said, there’re only two of us in the orchestra at the moment. The other… the actual principal wasn’t available. So that basically leaves just one person left. That’s me. Or, the management can decide that they do not want me to do it and they want to bring a guest in.*

Initially, Adam thought that the management’s final decision was to employ a guest performer for the occasion. Adam *appraised* the demand as a threat to his reputation in the orchestra: “What that communicates to me… they’d rather not have the risk of… using me, who’s on salary, so I do not cost them any more money… this will make me feel that you have, I have not got your trust.” Adam experienced the *underlying property of threat appraisal* self and other comparison and was concerned about evaluation both by management and his colleagues. Adam’s perception of management was that they had “a perceived hierarchy… and I do not fit into the… top tier.” In terms of evaluation by colleagues, Adam said, “in the paranoid part of my brain started thinking, blimey… has one of my colleagues said, ‘I’d rather not have Adam play this part’?” Adam used problem solving as a personal *resource* and decided to speak with management: “I had a little bit of a conversation with them, had to email them about this… yeah, it was a miscommunication but I had to communicate.” Adam implied that the situation had a negative impact on his *well-being*: “I felt I needed to communicate… what that… for my own confidence and my own mental health.”

## Discussion

This study sought to interpret the lived experiences of perceived occupational stress and well-being of professional classical musicians through understanding the demands faced, appraisals made, resources used, and the perceived influence on self-reported well-being. Each of these aspects is addressed in turn in the discussion below followed by consideration of the relationship between these elements of the stress process and well-being outcomes. The following sections are aligned to the five research questions outlined in the Introduction.

### Perceived demands (RQ1)

Participants discussed demands within the Group Experiential Themes: (a) Performance Demands; (b) Organizational Demands; (c) Relationship Demands. These themes relate to the categories of occupational demands experienced by classical musicians as identified in a systematic review by Vervainioti and Alexopoulos (2015). The theme Performance Demands included demands related to performance contexts, exposure, and performance standards. Given the centrality of performances to musicians, it is unsurprising that participants often discussed Performance Demands. Such demands occurred across a variety of settings including orchestral and chamber ensembles, recording sessions, and auditions, all of which have been considered in the literature (e.g., Parasuraman and Purohit, 2000; Brodsky, 2006; Lim, 2014; Kegelaers et al., 2022). Participants also discussed the demand of meeting high performance standards, which related to high self-expectations and the perceived expectations of others. Similarly, Creech et al. (2008) reported that self-doubt regarding the ability to meet high performance demands was a significant challenge for musicians.

Organizational Demands related to those demands controlled at the level of the organization (i.e., by management), which included touring schedules, travel, and role-related demands. Zendel (2021) suggested that the demands of touring, such as frequent travel and scheduling issues, could increase the precarity experienced by professionals. The experience of role-related demands might be due to the complexities of the work environment, and participants discussed role insecurity, role conflict, and role overload. Adam discussed two contrasting situations involving Role Demands. Firstly, Adam described performing across multiple roles within one organization. Here, Adam focused on the differences between the roles, which led to role conflict, which Anglin et al. (2022), Appendix C defined as “the occurrence of two or more incompatible behavioral expectations.” Secondly, Adam described presenting and performing within the same concert. However, this did not create role conflict and Adam viewed the roles as complementary. Adam’s experience is representative of the multiple roles musicians take on within a portfolio career and the findings suggest that role conflict may occur when roles are perceived as incompatible.

Relationship Demands related to interpersonal relationships with colleagues, peers, management staff, and audiences. Participants often discussed demands associated with colleagues, which may be due to the importance of such relationships for work opportunities and performance outcomes. Indeed, Dobson (2010a) discussed the term “professional sociability” and suggested that high interpersonal skills could lead to work retention and future work opportunities.

### Primary appraisals and underlying properties of stress appraisal (RQ2)

Across all demands experienced, threat appraisals were most commonly reported. A small number of demands were appraised as a challenge or benefit and the fewest appraisals were made for loss and harm. The frequency of threat appraisals suggests that the occupational environments of professional classical musicians can be characterized as threatening. Similarly, in a study of popular musicians, occupational demands were most commonly appraised as a threat (Cohen, 1999). However, Cohen (1999) reported that harm was the second most frequent appraisal, followed by challenge and benefit.

Threat appraisals frequently related to employment security, career advancement, and negative judgement by colleagues or management. The precarious employment conditions and threats to employment security that musicians experience have been discussed in the literature (Dobson, 2010a; Umney and Kretsos, 2015; Chafe and Kaida, 2019). Musicians often work on a freelance basis, which may lead to employment uncertainty alongside financial insecurity (Chafe and Kaida, 2019). That participants perceived a threat due to the potential for negative judgement by colleagues is also relevant to employment security. Coulson (2012) described the importance networking played in obtaining employment, with colleagues providing performance opportunities. Colleagues’ perception of performance skills is therefore crucial for musicians to be able to access employment.

Challenge appraisals were made when participants were in unusual circumstances but had experienced similar scenarios previously. This suggests an element of prior learning and reflection, where participants have previously benefitted from engaging with similar demands. Participants believed they could experience personal growth through taking on demands, which suggests they perceived the demands as an opportunity. Within CMRT, Lazarus (1999) suggested that perception of opportunity is part of the transactional stress process and contributes to challenge appraisal. Another potentially relevant concept is growth mindset, which is the belief that one’s traits are malleable and can be changed through effort (Dweck, 2008). Dweck and Yeager (2019) suggested that individuals with a growth mindset are more likely to seek out challenges, which may explain why participants made challenge appraisals when in unusual situations.

Benefit appraisals were made when participants perceived that the experience had been beneficial to their career, personal development, or audiences. Often, benefit appraisals were made after taking on new roles that represented career progression. Given the precarity of musical careers, many musicians adopt portfolio careers that encompass activities such as performance, teaching, and composing (Thomson, 2013). Portfolio careers may provide stability for musicians through regular engagements or teaching work, whilst also allowing time for performance work (Umney and Kretsos, 2015). Therefore, taking on new roles may have provided increased employment security and thus been perceived as beneficial.

Harm and loss appraisals related to not meeting the perceived role requirements and low-quality experiences. Consistent with CMRT (Lazarus, 1999), harm and loss appraisals were made when participants were unable to achieve their goals and perceived negative consequences. Harm and loss appraisals have been explored in high performance sports coaching (Didymus, 2017). Similarly to the present study, sports coaches reported harm or loss when they were unable to achieve their goals or perceived damage to their well-being.

Participants reported experiencing all 10 underlying properties of stress appraisal (Thatcher and Day, 2008). Two novel findings regarding underlying properties of stress appraisal emerged from this study. Firstly, participants discussed preparation more broadly than suggested by Thatcher and Day (2008), referring not only to inadequate preparation but also to adequate preparation. Secondly, considering self and other comparison, participants made direct as well as indirect comparisons.

Preparation was largely discussed in relation to practice and a significant body of literature exists on the topic of deliberate practice in music (e.g., Hambrick et al., 2014; How et al., 2021; Kegelaers et al., 2022). When participants discussed adequate preparation, they were more likely to report positive performance outcomes and the opposite was true for inadequate preparation, a finding reflected by Clark et al. (2014). A possible reason why adequate preparation was not suggested as an underlying property of stress appraisal by Thatcher and Day (2008) was that participants discussed their most stressful competition experience. Therefore, the situations considered are likely to have involved significantly high levels of demand. Contrastingly, in the present study, participants were asked to describe two demanding scenarios: one perceived negatively and one perceived positively. This may account for why adequate preparation emerged as an additional underlying property of stress appraisal.

The most common underlying property of stress appraisal was self and other comparison, which was observed in two ways. Firstly, participants made direct comparisons between themselves and colleagues or their past selves. Secondly, they compared themselves to a tacit industry standard, which caused them to be concerned about evaluation from colleagues. These can be considered as indirect comparisons and this type of self and other comparison is a novel finding of the present study. Considering direct comparison, participants compared themselves both favorably and unfavorably suggesting a balanced perspective. Self and other comparison is embedded in the careers of classical musicians, due to auditions and orchestral trial periods, which can last months or even years (Noden, 2017). Musicians are in direct competition in auditions, which may encourage individuals to compare their skills (Kegelaers et al., 2022). Considering indirect comparisons and the need to perform in line with professional standards, participants were concerned whether their performances were of a high enough standard and the potential for losing work due to poor performances. Similarly, self-employed musicians reported that musical ability was an important aspect of their reputation (Portman-Smith and Harwood, 2015). Freelance jazz musicians have also described a need to prove themselves during performance (Dobson, 2010b).

### Occupational and personal resources (RQ3)

Participants discussed using both personal and occupational resources to cope with the demands they experienced, frequently relying on personal resources. Personal resources included the use of psychological skills, problem solving, performance preparation, emotion regulation, and avoidance. Workplace resources included social support from colleagues, development opportunities, provision of autonomy, and organizational resources. Participants used resources that were appropriate to the type of demand they experienced: for instance, using psychological skills to address performance demands. In this section, the resources referred to most often are considered, including psychological skills, social support, and development opportunities.

Psychological skills used by participants included imagery, mental rehearsal, self-talk, cognitive restructuring, and mindfulness techniques. The results from a systematic review by Ford and Arvinen-Barrow (2019) suggested that psychological skills interventions are effective in supporting musicians to cope with demands and can lead to enhanced performance skills, reduced anxiety, and greater self-efficacy. Participants had developed their psychological skills through formal and informal learning: Adam had received cognitive-behavioral therapy (CBT); Daniel’s interest in meditation led him to use mindfulness techniques. Adam perceived the psychological skills he had learnt during CBT as effective for managing performance demands and continued to apply the techniques in performances.

Participants considered social support from colleagues, supervisors, and audiences. Colleague social support and its relevance to professional musicians has been examined in the literature (e.g., Dobson and Gaunt, 2015; Ascenso et al., 2017; Parker et al., 2019). In the present study, participants discussed different types of social support including emotional, esteem, informational, and tangible support, which aligns with the work of Cutrona and Suhr (1992). Popular musicians have reported using informational support in the form of sharing information and skills between band members (Vaag et al., 2014). Musicians also provide tangible support in the form of work opportunities for colleagues and accommodation (Coulson, 2012). Whilst the majority of the participants perceived that colleague social support was available, Daniel described a lack of colleague support and instead relied on his personal resources. Social support from supervisors and audiences was also discussed by participants although less frequently.

Development opportunities were also discussed as an organizational resource. These opportunities allowed participants to work in different settings. Some participants perceived a lack of development opportunities within their roles or that the available opportunities were inadequate. This may be due to the flat organizational structure of orchestras leading to limited opportunities for career progression.

### Well-being experiences (RQ4)

Participants reported both hedonic and eudaimonic well-being outcomes. They discussed acute emotional responses at the time of experiencing occupational demands (i.e., positive and negative affect) alongside long-term well-being outcomes (i.e., satisfaction and eudaimonic well-being), which aligns with CMRT (Lazarus, 1999). Considering positive affect, participants experienced enjoyment, excitement, inspiration, and pride due to their musical experiences. Similarly, Ascenso et al. (2017) reported that music-making was an important contributor to positive emotions in classical musicians. Participants also discussed negative affective outcomes in the form of somatic anxiety such as feeling discomfort. Participants reported acute anxiety due to performance scenarios and a large body of literature exists regarding MPA, its prevalence, and possible interventions (e.g., Kenny, 2011; Fernholz et al., 2019).

Participants described experiencing satisfaction and dissatisfaction as an outcome. Satisfaction was reported when performances had gone well, aligning with research which suggests making-music is itself a source of satisfaction for musicians (Coulson, 2012). Participants reported dissatisfaction when either their own performance or the actions of others failed to live up to their expectations. This is similar to research from the occupational literature, which suggests that unmet expectations may have a negative impact on job satisfaction (Murray, 2008; Irving and Montes, 2009).

Alongside hedonic well-being outcomes, participants reported experiencing all six dimensions of eudaimonic well-being. They frequently referred to environmental mastery, which was linked to performance outcomes: positive performance outcomes led to increased environmental mastery; conversely, negative performance outcomes led to decreased environmental mastery. Environmental mastery is similar to the concept accomplishment, which is part of Seligman’s (2011) PERMA framework and has been explored in musicians (Ascenso et al., 2017). The experience of environmental mastery may be due to the fulfilment or thwarting of competence, which is a basic psychological need within the framework of Self-Determination Theory (Deci and Ryan, 2000).

Personal growth was discussed by participants in relation to working in new settings and gaining new perspectives on themselves and their work. This finding is echoed in research on critical life events in sport and music where individuals have reported experiencing personal growth through increased maturity, greater self-understanding, and developing perspective on what was important (John et al., 2019). Purpose in life, self-acceptance, and autonomy were discussed less frequently by participants.

### Connections between stress and well-being (RQ5)

In this section, connections between the stress process and well-being outcomes are considered. Well-being outcomes related to the type of appraisal participants made. Threat and challenge appraisals related to acute hedonic well-being experiences. Threat was related to the experience of negative affective well-being outcomes and challenge was related to positive affective well-being outcomes. Researchers assessing the role of stress appraisals for employee well-being have reported similar findings. For instance, threat appraisals have been related to higher distress and anger, whereas challenge appraisals have been related to higher positive affect (Searle and Auton, 2015; Tuckey et al., 2015). Tuckey et al. (2015) suggested that threat appraisals may have a negative emotional impact due to the threat to basic psychological needs. Conversely, challenge appraisals may lead to positive affect due to the fulfilment of basic psychological needs.

Longer term well-being outcomes, including satisfaction and aspects of eudaimonic well-being, were related to appraisals of benefit and harm/loss. Satisfaction was also related to positive impacts on eudaimonic well-being experiences (e.g., increased environmental mastery) and was connected with appraisals of benefit. This often related to the effective use of resources, which was associated with successful performance outcomes. Harm and loss appraisals were connected with dissatisfaction and experiences that negatively impacted eudaimonic well-being (i.e., related to ill-being). Such experiences were associated with ineffective use of resources and negative performance outcomes. Additionally, harm and loss appraisals were associated with negative affective outcomes, such as anger, when participants perceived they had been wronged or mistreated. It is important to note the temporal element of appraisals and associated well-being outcomes: challenge and threat appraisals were made prior to events and affected acute well-being outcomes; benefit and harm/loss appraisals were made following events and led to lasting well-being outcomes.

### Implications

Regarding underlying properties of stress appraisal, the results demonstrate that “inadequate preparation” should be revised to “preparation,” as participants discussed being both adequately and inadequately prepared. Preparation could be defined as “the extent to which an individual feels prepared for performance.” Additionally, self and other comparison could be expanded to account for comparisons with perceived industry standards and be defined as “comparing any physiological, psychological, or social aspect of performance or the associated environment with that of another individual or perceived occupational standards.” The comparison to a tacit standard may reflect how classical music is taught. Smilde (2009) suggested that tacit knowledge is often communicated by an experienced individual in close proximity over a number of years. The reliance on tacit knowledge could mean that many aspects of the occupational environment remain unspoken (a hidden curriculum), which could explain why musicians compare themselves to a tacit industry standard rather than agreed and explicit standards.

Practical implications for professional orchestras relate to continuing professional development (CPD) and creating socially supportive environments. Given that participants made challenge appraisals when in new or unusual circumstances, CPD represents one way to provide such experiences. For orchestral musicians, this could be achieved through performing in smaller ensembles, programming concerts, or working in healthcare or educational settings. Findings from qualitative research suggest that working in educational and community contexts positively impacts musicians’ well-being and facilitates the development of new skills (Preti and Welch, 2013; Ascenso, 2016; Forbes and Bartlett, 2020a). For such opportunities to be appraised as a challenge rather than a threat, it is important they are adequately resourced. This means musicians need appropriate time, training, and support. For those working in healthcare settings, this could be training to understand specific conditions, introductions to specific models of working, or peer learning sessions (Perkins et al., 2018; Forbes and Bartlett, 2020b; Shaughnessy et al., 2023).

In terms of social support, orchestra managers can provide different types of support, such as informational support (e.g., feedback on performance), tangible support (e.g., finance to attend counselling), emotional support (e.g., talking to musicians about how they are feeling), and esteem support (e.g., acknowledging when tasks are done well; Cutrona and Suhr, 1992). For musicians who frequently work with the same ensemble, regular conversations with section principals, ensemble leaders, or orchestra managers could be established. This could allow musicians to receive feedback in a non-threatening environment and provide an opportunity to discuss CPD. Regular conversations could allow managers to build social connection with musicians, making them more comfortable to discuss issues when they do arise. The results also highlight the importance of social support from colleagues. Increasing musicians’ abilities to support each other could be achieved by delivering training on communication and collaboration skills through reflective exercises and group activities (e.g., Jungert et al., 2018). Research from the wider occupational literature suggests that such interventions may be effective for increasing colleagues’ abilities to support each other in terms of basic psychological needs, and, in turn, positively impact well-being and motivation (Slemp et al., 2021).

### Strengths, limitations, and future directions

This is the first qualitative study to explore the stress process and perceived well-being outcomes from the perspective of CMRT (Lazarus, 1999) in professional classical musicians. Further, this is the first qualitative study to explore appraisals and underlying properties of stress appraisal in this population. The relationship between primary appraisal and well-being outcomes highlights the role of the individual in the stress process and the importance of transactional approaches for assessing stress and well-being in classical musicians. Thatcher and Day (2008) based their underlying properties of stress appraisal on situations perceived as extremely stressful. The inclusion of demands perceived as both positive and negative allowed for further development of the underlying properties of stress appraisal.

However, we acknowledge the limitations of the current study, in which data were collected during the COVID-19 pandemic. To increase the relevance of the results to the broader occupational environment, participants were asked to consider their usual workplace experience, which relied on participants accurately remembering and representing their experiences. Further, due to the idiographic nature of the research the sample was small. Whilst consistent with IPA, the findings are based on the experiences of a few participants.

In future, researchers should consider asking participants to reflect on situations that they perceive both positively and negatively to elicit understanding of the stress process and well-being outcomes rather than focusing on experiences that are considered as detrimental to the individual. Additionally, researchers can use the expanded definitions for preparation and self and other comparison proposed in this study. Including not only inadequate preparation but preparation more broadly provided a better understanding of why different types of appraisal were made. Given the nature of IPA research and focus on small sample sizes, future research could be conducted with a large sample of musicians using quantitative methods. This could include cross-sectional or longitudinal questionnaire studies that assess the different aspects of the occupational stress and well-being process, and the relationships between these concepts.

Considering the evidence presented here, researchers could conduct interventional studies that address occupational stress and well-being in musicians. At an organizational level, interventions could target social support in the workplace and professional development programmes. Given that many musicians work in a freelance capacity, the music industry could create well marketed and funded networks for musicians that support them to develop the resources needed for a thriving career. At an individual level, further intervention studies are required to establish how professional musicians can be taught psychological skills as the majority of research has been conducted with students.

### Conclusion

This study took a qualitative approach to assessing occupational stress and well-being in professional classical musicians. Across occupational demands, threat appraisals were most frequent, which suggests that the occupational context of classical musicians can be characterized as threatening. The main threats perceived by participants related to career and employment security. Self and other comparison and preparation were two important underlying properties of stress appraisal. We provided updated definitions for both concepts. Participants described personal resources and organizational resources, often discussing social support. Through using resources, participants often described successful outcomes, particularly in terms of performance. Stress appraisals were seen to relate differentially to well-being outcomes. Threat and challenge appraisals, which are future orientated, related to acute hedonic well-being outcomes in the form of positive and negative affect. Benefit and harm/loss appraisals, which are orientated towards the past, related to satisfaction and eudaimonic well-being.

## Data Availability

The datasets presented in this article are not readily available because they are restricted to the author team in accordance with Cardiff Metropolitan University ethics committee. Requests to access the datasets should be directed to SW, WillisS5@cardiff.ac.uk.

## Footnotes

^1^ https://transcribe.wreally.com/
