# Supplementary material for: “I feel like a fish out of water”: interpreting the occupational stress and well-being experiences of professional classical musicians
Source: Front Psychol. 2024 Aug 14;15:1374773. doi: 10.3389/fpsyg.2024.1374773 (PMC11351566; doi:10.3389/fpsyg.2024.1374773)
Supplement: Supplementary file 1 [file Table_1.DOC]

Supplementary Material

Interview Guide

I am interested in the day-to-day working environment of musicians. I would like you to reflect back to the time before COVID-19 and keep this in mind as we talk. I recognise a lot has changed for us all and your current situation may be very different.

1. What type of work do you do?
2. What were the day to day demands you experienced?

Some parts of your job may be challenging whilst also providing opportunities for growth and development. Other aspects of your job may be more demanding and might have negative or distressing outcomes for you or those around you.

**Negative Demand Experience**

1. Did you experience any aspects of your career experience as very demanding?
2. Can you give me a specific example of a *relatively recent* (pre-COVID-19) situation that was very demanding? (One that might have had a more negative outcome or been more distressing)
3. Considering that situation, what were your thoughts about it at the time?
4. Thinking about that situation, how did feel you at the time?
5. What did you do to cope with that situation?
6. What was the outcome of that situation?

**Positive Demand Experience**

1. Were there any aspects of your job that created opportunities for you to develop or grow?
2. Can you give me a specific example of a *relatively recent* (pre-COVID-19) situation that was challenging but also provided you with an opportunity to develop?
3. Considering that situation, what were your thoughts about it at the time?
4. Thinking about that situation, how did feel you at the time?
5. What did you do to cope with that situation?
6. What was the outcome of that situation?

**Support**

1. Do you know of any help or support that is available to musicians to help them cope?
2. Is there any help or support that you would like to be available to musicians?
3. Is there anything else you’d like to discuss today?


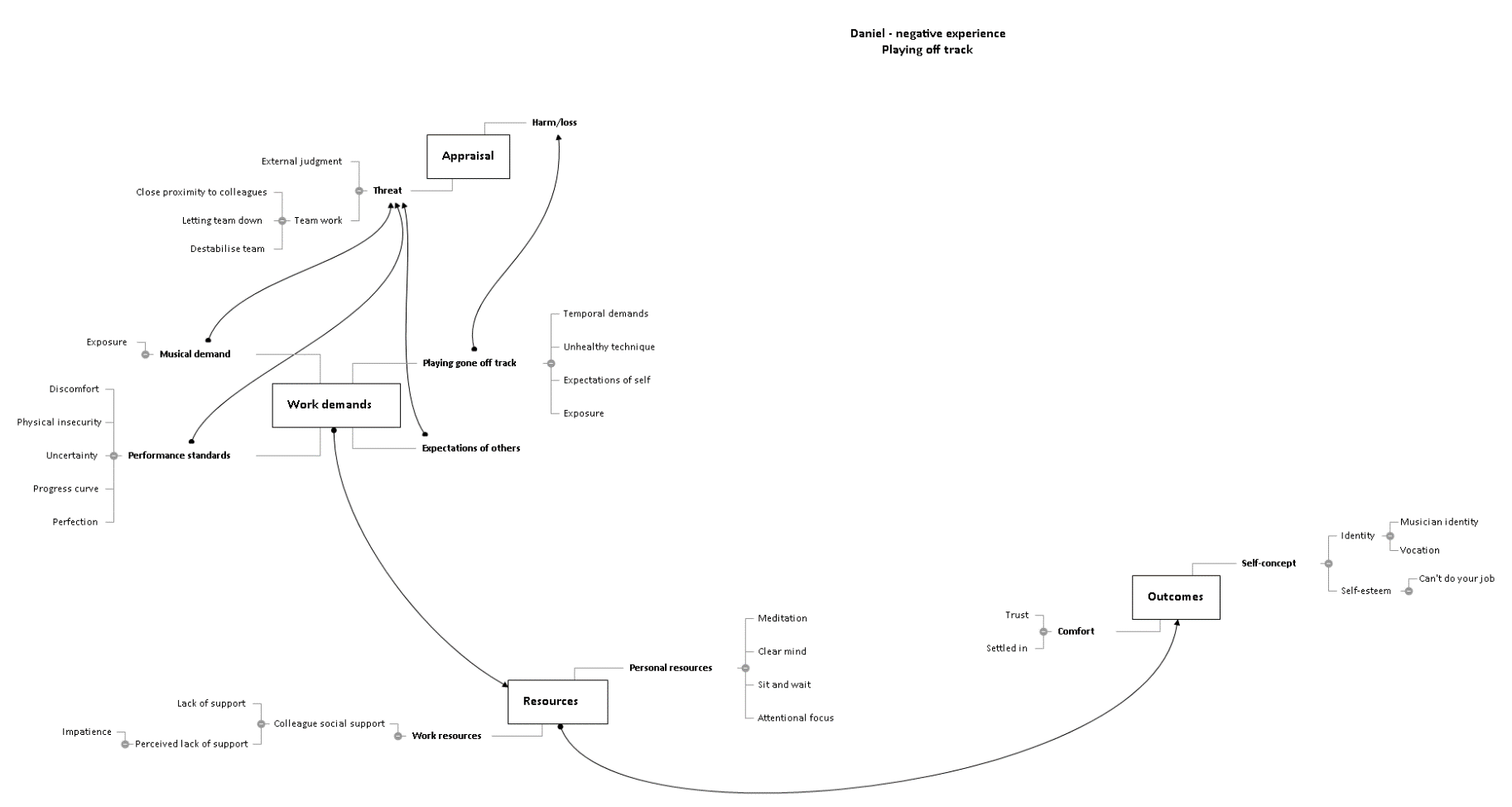


**Supplementary Figure 1.** Example of Personal Experiential Themes. Daniel’s negative experience

Summary of professional classical musicians’ occupational stress experiences

| **Demand** | **Participant** | **Demand theme** | **Appraisal** | **Underlying Properties** | **Personal resource** | **Occupational resource** | **Hedonic well-being** | **Eudaimonic well-being** |
| --- | --- | --- | --- | --- | --- | --- | --- | --- |
| Multiple roles (chamber) | Adam | Organisational | Threat | Temporal uncertainty | Psychological skills | − | − | − |
| Multiple roles (presenting) | Adam | Organisational | Challenge | Novelty Comparison | − | − | − | − |
| Organisation (tour) | Ben | Organisational | Threat | Predictability Event uncertainty Imminence | Problem solving | − | Negative affect | − |
| Travel (tour) | Ben | Organisational | Threat | Duration Temporal uncertainty | Problem solving | − | − | − |
| Competing employment | Ben | Organisational | Threat Benefit | Predictability Imminence | Problem solving | − | − | − |
| Travel (audition) | Ben | Organisational | Threat | Temporal uncertainty | Problem solving | − | Negative affect | − |
| Competing employer interests | Charlotte | Organisational | Threat | Predictability Event uncertainty | Problem solving | − | − | Mastery |
| Organisation | Charlotte | Organisational | Threat | Preparation | − | − | Negative affect | − |
| Responsibility to clients | Charlotte | Organisational | Threat | Event uncertainty | Problem solving | − | − | − |
| Travel | Charlotte | Organisational | Threat | Predictability Duration Temporal uncertainty | Problem solving | − | Negative affect | − |
| Work conditions | Charlotte | Organisational | Threat | Predictability | Problem solving | − | Negative affect | − |
| Role criteria | Daniel | Organisational | Threat | Comparison | − | Autonomy | − | − |
| Leadership | Eva | Organisational | Threat | Comparison | − | − | Negative affect | − |
| Schedule | Eva | Organisational | Benefit | Imminence | Psychological skills | Social support | − | − |
| Travel | Kieran | Organisational | Threat | Duration | − | − | Negative affect | − |
| World tour | Kieran | Organisational | Threat Challenge Benefit | Novelty Event uncertainty | − | Social support Development opportunities | Positive affect | Growth Mastery |
| Devolved responsibility | Kieran | Organisational | Threat | Predictability | − | − | Negative affect | − |
| Responsibility for orchestra | Kieran | Organisational | Threat | Event uncertainty | − | − | Negative affect | − |
| Exposure | Adam | Performance | Threat  Benefit | Novelty Duration Comparison | Psychological skills | Social support | Negative affect | − |
| Performance standards | Adam | Performance | Threat | Event uncertainty | Psychological skills | Social support | − | Mastery |
| Presenting | Adam | Performance | Challenge Benefit | Comparison | Preparation | Social support | Positive affect | − |
| Audition | Ben | Performance | Threat Benefit | Novelty Duration Preparation | Psychological skills Physiological | Social support | Positive Affect Negative Affect Satisfaction | Mastery |
| Performance standards | Ben | Performance | Challenge | Event uncertainty | Preparation Emotion regulation | − | − | − |
| Musical | Ben | Performance | Threat Challenge | Novelty Imminence Comparison | Psychological skills | Social support | Positive affect | Mastery Purpose |
| Subsequent performance with orchestra | Ben | Performance | Threat | Comparison | − | − | Negative affect | − |
| Arranging music | Charlotte | Performance | Threat Benefit | Duration | Passion Problem solving | Development opportunities | − | Growth Purpose |
| Performance demand | Charlotte | Performance | Threat | Comparison Preparation | Physiological | − | Negative affect | − |
| Chamber music | Daniel | Performance | Threat Benefit | Duration Comparison Preparation | − | Social support Autonomy Organisational resources | Positive Affect Negative Affect Satisfaction | − |
| Exposure | Daniel | Performance | Threat | Event uncertainty Comparison | − | − | Negative affect | − |
| Musical | Daniel | Performance | Threat | Event uncertainty Comparison Preparation | Preparation | − | Negative affect | Mastery |
| Performance standards | Daniel | Performance | Threat | Comparison Preparation | Psychological skills | Social support | Negative affect | Mastery |
| Interpretational difference | Eva | Performance | Threat Harm | Ambiguity Comparison | Psychological skills | Social support | Negative affect | Acceptance |
| CD recording | Eva | Performance | Challenge  Benefit | Duration Preparation | − | Social support Autonomy | Positive Affect Negative Affect | Autonomy Growth |
| Performance spontaneity | Eva | Performance | Threat | Predictability | − | − | Negative affect | − |
| CD edit | Eva | Performance | Threat | Comparison | Emotion regulation Understanding self and coping | Autonomy | Negative affect | − |
| Televised performance | Eva | Performance | Threat | Comparison | − | − | − | − |
| Mobile phone concert | Kieran | Performance | Challenge | Novelty | Psychological skills | − | Positive Affect Negative Affect | Purpose |
| Unusual conducting setup | Kieran | Performance | Threat | Novelty | − | − | Negative affect | − |
| Unusual notation | Kieran | Performance | Challenge | Novelty | − | − | − | − |
| Management miscommunication | Adam | Relationship | Threat | Comparison | Problem solving | − | Negative affect | − |
| Colleagues’ status | Adam | Relationship | Threat | Comparison | Preparation | − | − | − |
| Controlling emotions | Ben | Relationship | Threat | Comparison | Psychological skills | − | Positive affect | − |
| Audition feedback | Ben | Relationship | Threat | Comparison | Psychological skills | Social support | Negative affect | − |
| Colleague argument | Charlotte | Relationship | Threat Loss Harm | Novelty Predictability | Physiological Emotion regulation | − | Negative affect | Relationships |
| Dementia work | Charlotte | Relationship | Benefit | Preparation | Psychological skills | Development opportunities | Positive Affect Negative Affect | Purpose Growth Relationships |
| Sensitivity to clients | Charlotte | Relationship | Benefit | Event uncertainty | − | Social support | − | − |
| Working with vulnerable people | Charlotte | Relationship | Threat Loss | Event uncertainty Preparation | Emotion regulation | Social support | Negative affect | − |
| Client disclosure | Charlotte | Relationship | Harm | Preparation | − | Social support | Negative affect | − |
| Controlling emotions | Charlotte | Relationship | Threat | Comparison | Escape | − | − | − |
| Follow-up communication | Charlotte | Relationship | Harm | Novelty Predictability Comparison | Escape | − | Negative affect | − |
| Future interactions with colleague | Charlotte | Relationship | Threat | Event uncertainty | − | − | Negative affect | − |
| Apathy from musicians | Kieran | Relationship | Threat | Comparison | Problem solving | − | − | − |
| Relationships with colleagues | Kieran | Relationship | Threat Loss | Duration Ambiguity | − | Social support |  | Relationships |

Note. Comparison = self and other comparison
